# The Effects of Glutamine Supplementation on Reducing Mortality and Morbidity among Burn Patients: A Systematic Review and Meta-analysis of Randomized Controlled Trials

**DOI:** 10.1016/j.jpra.2022.09.003

**Published:** 2022-10-09

**Authors:** Hatan Mortada, Nawaf Alhindi, Abdulrahman Abukhudair, Shahad Alanazi, Alaa AlSahli, Khalid Arab

**Affiliations:** a Division of Plastic Surgery, Department of Surgery, King Saud University Medical City, King Saud University and Department of Plastic Surgery & Burn Unit, King Saud Medical City, Riyadh, Saudi Arabia; b Faculty of Medicine, King Abdulaziz University, Rabigh, Saudi Arabia; c Division of Plastic Surgery, King Abdulaziz University Hospital, Jeddah, Saudi Arabia; d College of Medicine, King Saud bin Abdulaziz University for Health Sciences, Riyadh, Saudi Arabia; e Division of Plastic Surgery, Department of Surgery, College of Medicine, King Saud University, Riyadh, Saudi Arabia

**Keywords:** Glutamine (GLN), critically ill patients, nutrition, burn, meta-analysis

## Abstract

Glutamine (GLN) has been proven to improve the prognosis of severely burned patients. GLN supplementation in critical illness has gained extreme popularity among researchers over the years, and its safety and efficacy are still under question. Therefore, we aim to study the role of GLN supplements in decreasing mortality, length of hospitalization (LOH), and infection in severely burned patients. PRISMA guidelines were used to design and conduct this systematic review. MEDLINE, Cochrane, and EMBASE databases were used to search for randomized controlled trials (RCTs) in January 2022. In order to assist in the search, MeSH terms such as burn injury, GLN, and RCT were used. As a result of reviewing the literature, 1112 publications were found. We included only 7 RCTs after implanting our inclusion criteria. There were 328 patients enrolled in the study, with 166 patients (50.61%) were allocated to GLN supplementation and 162 patients in the control groups (49.39%). The risk of infection was significantly lower among patients who received GLN supplementation than those in the control groups (RR = 0.41, 95% CI, 0.18 to 0.92, *p* = 0.030). The risk of death was significantly lower among GLN-receiving patients compared to non-GLN-receiving patients (RR = 0.09, 95% CI, 0.01 to 0.63, *p* = 0.016). GLN supplementation has been linked to lower hospital mortality and infection-related morbidity in burn patients. Furthermore, larger-scale and higher-quality studies are needed to assess whether there are any statistically and clinically significant changes.

## Introduction

*Burns* are devastating injuries affecting the human body. Burn victims face massive stress and tend to develop complications due to the significant impact on their body's physiologic and immunologic function, fluid, and nutrition.^1^, ^2^, ^3^, ^4^ Glutamine (GLN) is known to be the most abundant and versatile (nonessential) amino acid under normal healthy status. It contributes as a substrate to the production and synthesis of glutathione and ammonia, which are essential for all cellular replication.^5^ Nonetheless, GLN is known to be dramatically deficient in critically ill individuals, including burn victims. This deficiency is explained by increased body requirements exceeding production in response to the stressful status and catabolic events. These findings indicate that GLN has a significant role in such severely ill patients.^6^, ^7^, ^8^, ^9^ GLN supplements in critical illness have gained extreme popularity among researchers over the years, and their safety and efficacy are still under question. Many systematic reviews showed that GLN supplements effectively reduced mortality and complications such as gram-negative bacterial infection.^10^, ^11^, ^12^

Furthermore, a meta-analysis conducted in 2015 found that enteral GLN supplementation is more effective among burn patients than trauma and nonburn intensive care unit (ICU) patients in reducing mortality and length of hospitalization (LOH), with no difference in infectious mortality.^12^ However, over the past six years, new multicenter clinical trials have revealed that GLN supplementation, either parenteral, enteral, or in combination, is essential in early postburn management as it protects vital organs like the heart, preserves the intestinal mucosal thickness, and alleviates the hyper-metabolic status, which prevents further loss of the muscular bulk.^13^^,^^14^ The majority of previous systematic reviews and meta-analyses identified the efficacy of GLN in critically ill patients in the ICU and oncology patients, postabdominal surgery, and burn units. However, the most recent meta-analysis focused on burn patients was in 2012.^10^ Even though they have been shown to be the most beneficiary group from GLN supplements, they have not been considered in the most recent trials over the last ten years. This analysis aimed to study the role of early GLN supplementation on the body's systems, nutrition, and metabolism in preventing infection in severely burned patients. Also, the role in decreasing mortality, morbidity, and LOH.

## Methods and Materials

### Search Strategy

This systematic review and subsequent meta-analysis were carried out following the Preferred Reporting Items for Systematic Review and Meta-Analysis (PRISMA) guidelines.^15^ The following online databases were searched from inception to January 22, 2022. This analysis aimed to identify related randomized clinical trials (RCTs) from MEDLINE, Cochrane, and EMBASE. They were searched using the following keywords to aid the search: ``burn OR burn injury OR thermal injury'' AND ``glutamine OR glutamine dipeptide OR L-alanyl-L-glutamine OR parenteral nutrition'' AND ``RCT OR randomized controlled trial OR clinical trials''. We strived to review available published literature that reported the results of GLN supplementation in adult burn patients to determine its influence on mortality, morbidity, and LOH. The International Prospective Register of Systematic Reviews was utilized in this review on February 17 and identified as (CRD42022304655).^16^ This article adheres to the guidelines established via the Declaration of Helsinki in 1975.

### Study selection

Initial screening of articles by title and abstract was conducted by two independent groups consisting of four authors each (A.S and N.A) and (S.A and A.A), and the fifth author (H.M) resolved any conflict of inclusion in both groups. Related articles underwent further analysis by full text to ensure relevance and applicability. Inclusion of articles was limited to: (1) articles reporting randomized clinical trials (RCTs); (2) published from inception up to Jan 2022; (3) reported in English; (4) adult male and female patients above 18 years old; (5) the sample consisted of more than 10; and (6) reported the outcomes of burn patients who received either parenteral or enteral GLN supplement.

Meanwhile, studies were excluded if they met one or more of the following criteria: (1) language other than English; (2) reported a systematic review, case report, economic analysis, animal or cadaveric studies, retrospective, cohort, and cross-sectional studies; (3) used an intravenous route of GLN supplement; and (4) used non-GLN supplements.

### Data extraction

The included articles were extracted and reviewed by two authors (A.S and N.A), covering critical data such as intervention details (GLN route, dose, and supplement duration), sample size, and demographic data of the samples (gender and age), the sample characteristics or severity of the injury (burn type, total body surface area (TBSA%), and burn index), laboratory findings (albumin, creatinine, and glucose) level, and the hospital course (length of ICU stay, LOH, duration of mechanical ventilation, hospital mortality, and infection rate).

### Bias assessment

Using the Cochrane risk-of-bias tool for randomized trials (RoB 2), RCTs were assessed for bias.^18^ All study categories were evaluated for randomization, allocation concealment, blinding of participants and employees, blinding of observers, incomplete data, and selective reporting, with each category receiving a ``low risk,'' ``high risk,'' or ``some concern'' rating.

### Statistical Analysis

Statistical analysis was carried out using RStudio (R version 4.1.1). The frequencies of selected outcomes (suspected infection, confirmed infection, and mortality) were collected, and the pooled estimates were quantified using risk ratios (RRs) and their respective 95% confidence intervals (95%CIs). These meta-analytical models were constructed using the metabin package in R software. Regarding the length of hospital stay, the available records were collected as means and standard deviation, and the meta-analysis model was carried out using the metacont package. The result of the numerical variable was expressed as mean difference (MD) and 95%CI. Heterogeneity assessment was assessed using the I^2^ test. Fixed-effects models were implemented when there was no evidence of statistical heterogeneity (I^2^ < 50%); otherwise, random-effects models were constructed. In the instance of significant heterogeneity, we performed a sensitivity analysis by removing a single study from the model at once. Subgroup analysis was not performed because the number of eligible comparisons was small. Publication bias was assessed visually by the interpretation of funnel plots, whereas the Egger's test was utilized to investigate the publication bias statistically. A *p*-value of 0.05 was considered to indicate statistical significance.

## Results

### Findings in the literature

**Figure 1:**
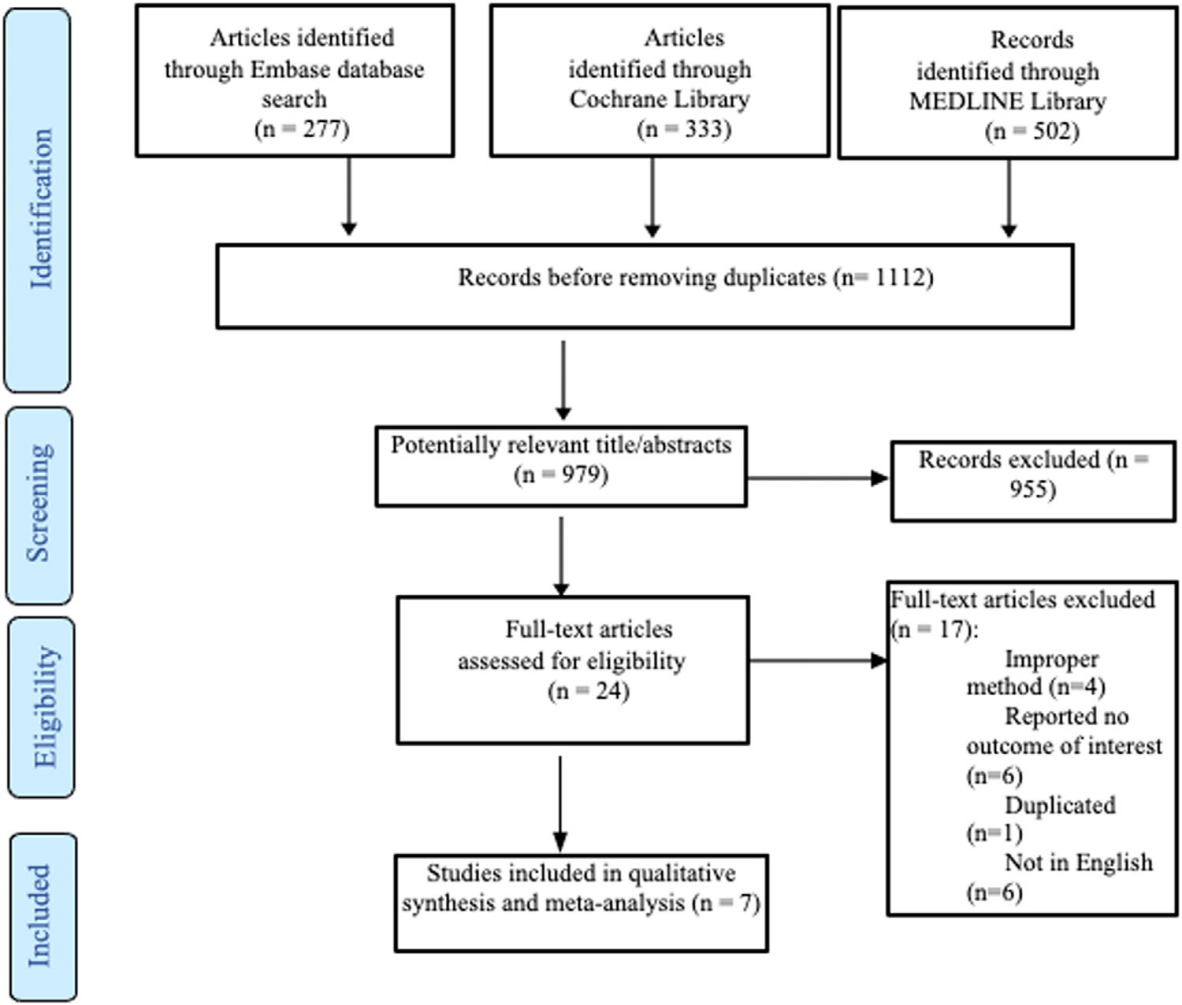
PRISMA flow chart for the systematic review.

Based on the results of this systematic review, 1112 published articles were found, including 277 articles from Embase, 502 articles from Medline, and 333 articles from Cochrane. The number of articles remaining for review was 979 after removing duplicates. A total of 24 full-text publications were initially retrieved. Nevertheless, only seven articles published between 2002 and 2021 were included after implementing the previous exclusion criteria (Figure 1). A total of seventeen articles were excluded due to the following reasons: Improper methods (systematic review, review article, letter to editor, and case report) (n = 4), no outcome of interest was reported (n = 6), duplicated (n = 1), and the full text was not in English (n = 6). An overview of each article is presented in Table 1. A total of seven RCTs were included in the present study. One study was published in North America^16^, one study in Europe^17^, and the remaining studies were published in Asia.^13^^,^^18^, ^19^, ^20^, ^21^, ^22^ These studies recruited a total of 328 patients (sample sizes ranged between 30 and 84 patients), of whom 166 patients (50.61%) were allocated to GLN supplementation and 162 patients in the control groups (49.39%). The studies were published between 2002 and 2021. Table 1 summarizes the remaining study characteristics.

**Table 1:** Characteristics of the included studies (C: control; G: glutamine; M: Male; F: female; TBSA: Total body surface area; NA: nonavailable)

Authors	Country	Sample size (G/C)	M/F	Age, mean ± SD		Characteristics of burn				Glutamine supplementation	
				G	C	Burn type	Inhalation injury	TBSA %	Burn index	Intervention dose, g/kg/day (method of administration)	Duration of the intervention
Garrel et al. 2003^16^	Canada	41 (22/19)	37/4	38.0 ± 7.0	38.0 ± 18.0	N/A	yes	C:42 ±16, G:40 ±18	97 ± 20 and 87 ± 27 for C and G, respectively.	26 g/day (Enteral)	At least 10 days
Griffiths et al. 2002^17^	UK	84 (42/42)	NA	NA	NA	N/A	N/A	N/A	N/A	17-24 g/d (Parenteral)	At least 5 days
Pattanshetti et al. 2009^22^	India	30 (15/15)	9/21	33.9 ± 0.0	29.1 ± 0.0	All types of thermal injury	Yes	20-60%	N/A	0.5 gm/kg/day (Enteral)	Till complete wound healings or after skin grafting
Peng et al. 2004^20^	China	48 (25/23)	29/19	NA	NA	N/A	no	30 - 75%	N/A	0.5 g/kg day of G (Enteral)	14 days
Wang et al. 2021^13^	China	55 (27/28)	43/12	NA	NA	(Flame:36, Scald:11, other:8)	Yes	30-70%	(Control:19.65-50.31, G group:24.96-56.16)	0.5 g/kg/day (Parenteral)	14 days
Zhou et al. 2003^19^	China	40 (20/20)	NA	43.7 ± 3.8	40.0 ± 4.3	Flame	no	50 - 80 %	APACHE II: C: 7.6 ±2.7 and G: 7.5 ± 1.8	0.5 g/kg/d (Enteral)	12 days
Zhou et al. 2004^21^	China	30 (15/15)	NA	34.6 ± 7.8	33.4 ± 8.1	N/A	no	30 - 50 %	APACHE II: C: 7.8± 2.1, G: 8.2±1.9	0.35 g glutamine kg bw/d (Parenteral for 16-20 h a day)	12 days

### Outcomes Ineligible for the Meta-Analysis

In two studies, liver function indicators were not significantly different between GLN-receiving and non-GLN-receiving subjects.^13^^,^^16^ Glucose concentration was reported in one study^1^, with no significant difference between the groups (8.6 ±1.4 in the GLN group and 8.9 ±1.8 mg/dl in the control group). Additionally, the duration of mechanical ventilation was similar between patients in the GLN and control groups (22 ± 10 and 24 ± 11 h, respectively). Creatinine and urea nitrogen concentrations were not reported in the included studies.

### The Results of the Meta-Analysis

**Figure 2:**
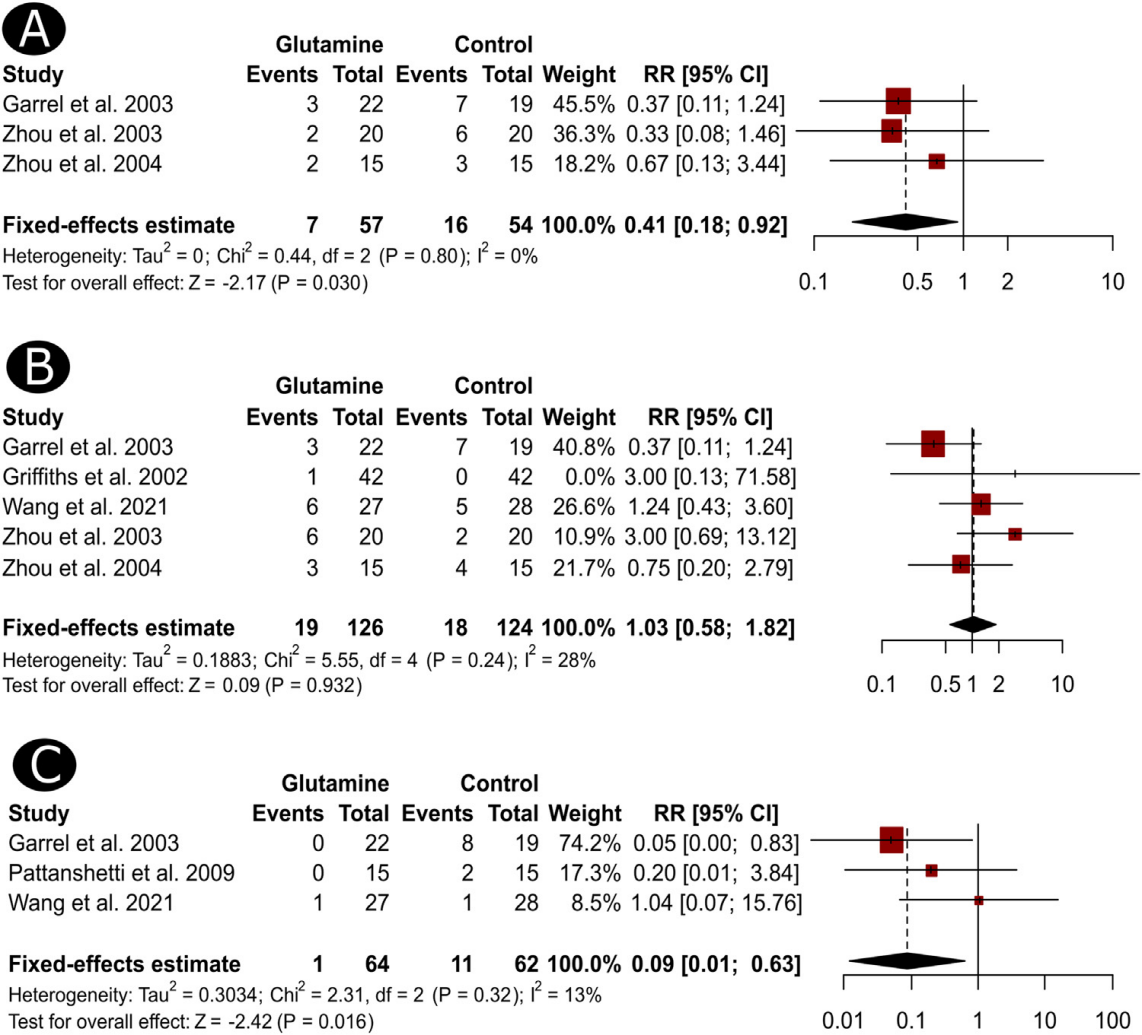
Forest plots depict the difference in the risk of suspected infection (A), confirmed infection (B) and mortality (C) between burn patients who received GLN and those who did not receive GLN.

Eligible comparisons for the meta-analysis were primarily related to the outcomes that have been reported in at least three studies. These included suspected infection, confirmed infection, mortality, and the LOH. The frequencies of patients with suspected infection were reported in three studies that recruited 111 patients.^16^^,^^18^^,^^22^ The risk of suspected infection was significantly lower among patients who received GLN supplementation than those in the control groups (RR = 0.41, 95% CI, 0.18 to 0.92, p = 0.030, Figure 2A). Nevertheless, based on the outcomes of five studies (n = 250) (16-18,22,13), the risk of confirmed infections was not significantly different between the GLN and control groups (RR = 1.03, 95% CI, 0.58 to 1.82, *p* = 0.932, Figure 2B). Interestingly, the risk of death was mentioned in three studies involving 126 patients.^13^^,^^16^^,^^22^ The pooled RR was significantly lower among GLN-receiving patients compared to non-GLN-receiving patients (RR = 0.09, 95% CI, 0.01 to 0.63, *p* = 0.016, Figure 2C). Of note, there was no significant heterogeneity between the studies in the outcomes of suspected infections (I^2^ = 0%, *p* for heterogeneity [h] = 0.800), confirmed infections (I^2^ = 28%, ph = 0.240), and mortality (I^2^ = 13%, ph = 0.320).

**Figure 3:**
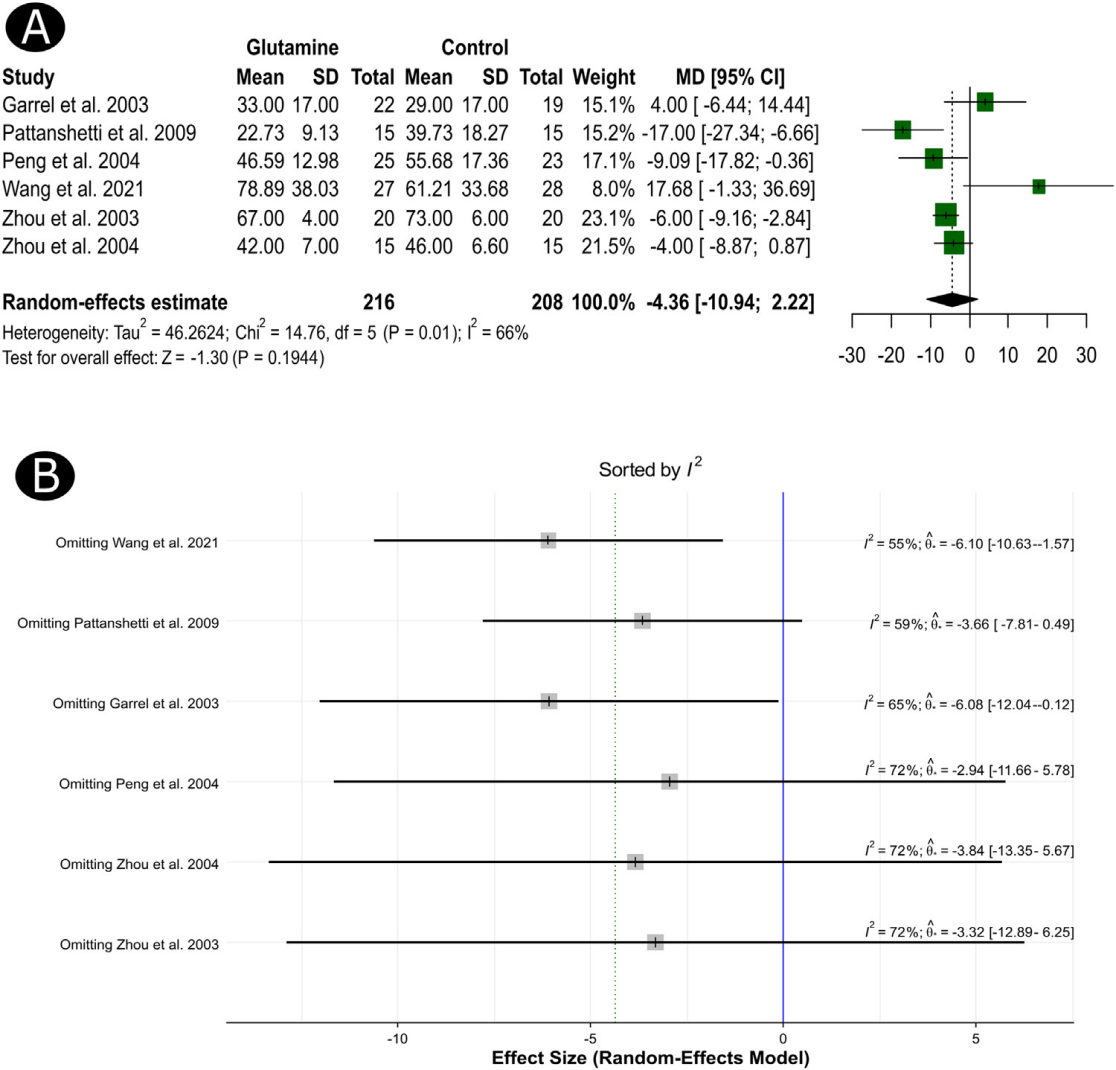
A forest plot shows the MD in the length of hospital stay (A) and the results of the influence analysis (sensitivity) for the same outcome (B).

Regarding the length of hospital stay, the individual results were reported in six studies (n = 424). The pooled MD was not statistically different among the GLN and control groups (MD = -4.36, 95%CI, -10.94 to 2.22, *p* = 0.194, Figure 3A). These results were based on a random-effects model owing to the significant heterogeneity between the studies (I^2^ = 66%, ph = 0.010). To further investigate the source of heterogeneity, we conducted a sensitivity analysis of the studies included in this particular outcome. Results indicated no distinct influential studies that impacted the statistical heterogeneity (I^2^ values remained above 50%); however, results revealed a significantly shorter length of hospital stay in the GLN arm than in the control groups, excluding the study of Wang et al.^13^ (MD = -6.10, 95%CI, -10.63 to -1.57) and Garrel et al.^1^(MD = -6.08, 95%CI, -12.04 to -0.12, Figure 2B).

### Publication Bias

**Figure 4:**
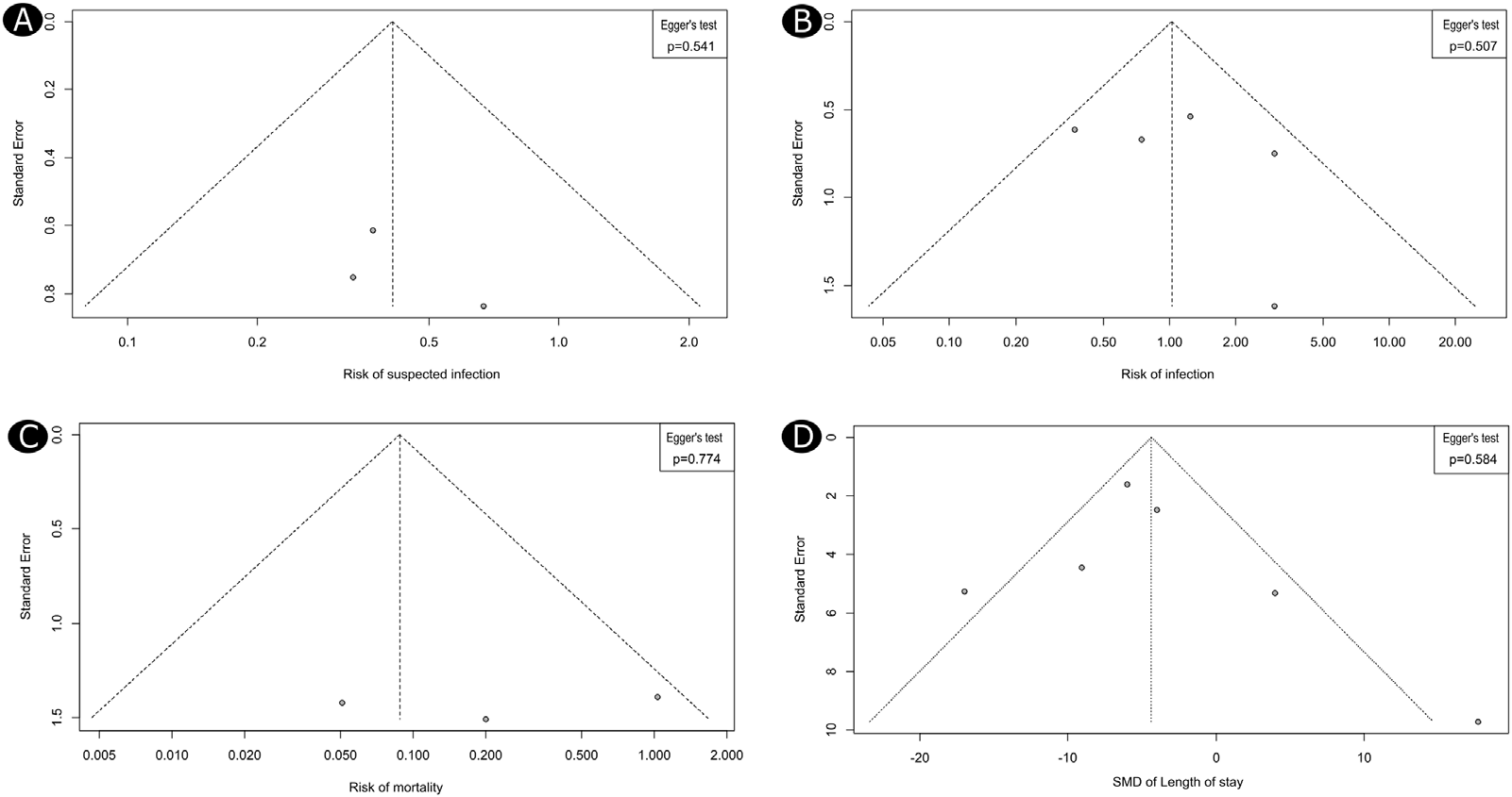
Funnel plots show the risk of publication bias across different outcomes in the meta-analysis, including the suspected infection (A), confirmed infection (B), mortality (C), and the LOH (D).

The risk of publication bias was assessed in four outcomes investigated in the meta-analysis, including the risks of suspected infection, confirmed infection, mortality, and the MD in length of hospital stay. Visual inspection of the funnel plots showed no significant asymmetry in the distribution of individual studies around the main effect estimate, which indicates no significant publication bias (Figure 4). This was confirmed statistically by the results of the Egger's test (p > 0.05 for all the outcomes, Figure 4).

*Quality Assessment and Risk of Bias*

**Figure 5:**
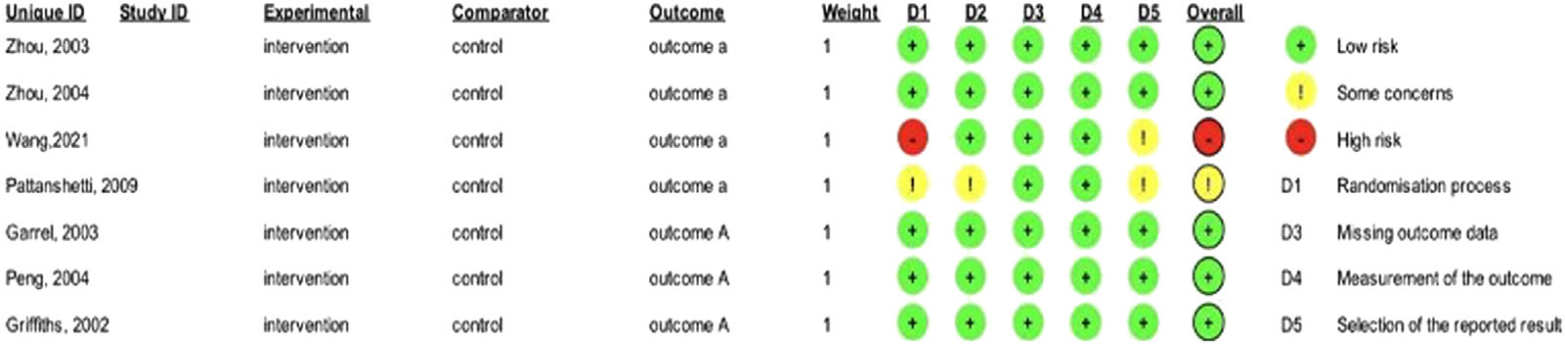
Risk of bias assessment summary

The risk of bias assessment of eligible RCTs was done independently by two reviewers using the Cochrane Risk of Bias Assessment Tool for Randomized Trials (RoB 2). The result shows that one study had a high risk of bias^13^, one study had some concerns about the risk of bias^22^, and the other five studies showed a low risk of bias (Figure 5).

## Discussion

Burns are known to have a significant impact on the human body, and the severity of the insult determines the LOH and mortality. The severity can be determined by different measures like the degree of burn, TBSA%, and the presence of comorbidity. However, the course of management can also influence the patient's LOH and outcomes. Nutritionists play a significant role in compensating the patient's body needs and restoring the physiologic body function. Burn patients have been found to gain much more benefit from GLN supplements than their counterparts, as reported by Van Zanten et al.^12^ Our overall findings state that GLN supplements can enhance a patient's survival and reduce the mortality rate. Even though we found that GLN reduced the rate of suspected infection, there was no significant difference in the confirmed infection rate, and LOH was almost the same as the control. Previous literature showed that GLN concentrations in intracellular and extracellular compartments decreased following injury.^23^ In the thermally injured, the association between GLN's low concentration and immunological function was studied in vitro by Parr-Billings et al. They showed that macrophages or lymphocytes' immunological function was reduced when exposed to low GLN concentrations.^23^^,^^24^ It was hypothesized that the provision of exogenous GLN could restore normal immunological function. This hypothesis was first tested by Ziegler et al.^25^ They provided posthigh-dose chemotherapy and radiation therapy with parenteral GLN during bone marrow transplantation. They found a 3-fold reduction in the occurrence of infection and a reduction in the length of hospital stay. Recently, systematic reviews and meta-analyses confirmed these findings in severely critically ill and burn patients.^10^, ^11^, ^12^ This study examined the role of GLN supplements in decreasing mortality, LOH, and infection in severely burned patients. Our present investigation showed a significant reduction in the risk of suspected infection among the GLN supplemented group compared to those in the control group. Also, there was no significant difference between both groups regarding confirmed infections. The mechanism of this phenomenon is unclear. As a precursor of glutathione, GLN is believed to have a significant antioxidant property, particularly on the gut mucosa.^26^ Previous animal studies suggest that GLN supplementation prevents bacterial translocation from the intestinal lumen to the bloodstream.^26^ Thus, it exerts a protective effect on the gut mucosa. However, bacterial translocation is still a debatable mechanism of nosocomial infections in humans.^27^ Another important finding of our study is the significant reduction in death rates among the GLN-supplemented group. The previous meta-analysis confirmed the same.^28^ The reduction of infection susceptibility could explain this finding. In their prospective randomized clinical trials ^27^, Dominique Garrel et al. demonstrated the reduction of blood culture positivity, particularly with pseudomonas aeruginosa, which is a major cause of mortality and morbidity in burn and ICU patients.^29^^,^^30^ This outcome was not likely to be biased regarding the provided care, as their study was blinded. Moreover, in terms of the severity of the injury, the TBSA and the incidence of inhalational injury were almost similar in both groups. However, in our included studies, the severity of burns was variable in all studies, as shown in Table 1. A previous meta-analysis that was conducted in 2012 showed that GLN supplementation reduced the length of stay. In contrast, six of our included studies reported the length of hospital stay, showing a nonsignificant pooled difference among both groups. The heterogeneity of this result was tested and showed distinct influential studies. However, results revealed a significantly shorter length of hospital stay in the GLN arm than in the control groups, excluding the studies of Wang et al.^13^ and Garrel et al.^1^ The impact of GLN on reducing LOH could be explained by its effect on factors associated with wound healing. Zhou et al. assessed wound healing 30 days postburn and found an improvement of only 19% in wound closure in the GLN supplemented group.^20^ However, it is still unknown whether this effect was due to the role of GLN in supporting protein synthesis, the support of inflammatory responses, or the general improvement in the health status of the supplemented group. The effect of GLN supplementation on liver function parameters was examined in only two of the included papers, showing no difference between GLN and non-GLN groups. This could be attributed to GLN's damaging and protective effects on the liver that form spontaneously through its complex metabolism and ``double-edged sword'' effect. It aggravates liver injury through its metabolic product NH4+^31^ and improves it by enhancing the liver's blood perfusion and inhibiting inflammation caused by bacteria and endotoxin translocation.^32^^,^^33^ Also, we suggest assessing liver indices before supplying the patient with GLN, especially in patients with severe burns. The effect of GLN supplementation on glucose levels was the only study included and showed no differences among both groups. A previous systematic review measuring the effects of GLN supplementation on metabolic variables in diabetes mellitus showed that it could decrease fasting blood glucose, postmeal glucose, and triglyceride levels and increase insulin production.^34^ Moreover, previous studies conducted on diabetic animals to measure the effect of GLN on glycemic status contradicted previous results. This was mainly due to the difference in duration of supplementation as well as the wide range of GLN dosage.^35^ In recent studies, GLN caused a significant reduction in glucose levels after four weeks of a 4.5 mg\kg GLN supplementation regime.^36^^,^^37^ Compared to the previously published meta-analysis^10^^,^^12^, the strength of this meta-analysis is the inclusion of all recent randomized controlled clinical trials over the past decade. Another point is the use of different methods to decrease bias: publication bias was considered in four outcomes; visual inspection was performed for the funnel plot; a complete search of the wide-reaching literature; and the removal of duplicate data. In contrast, a limitation of our study is the limited number of trials included. Based on that, subgroup analysis could not be carried out. It is also important to note that most of the trials did not include the details of the intensity of the complications and the overall morbidity rate, which could have produced a likely bias in our findings. For further studies, we recommend conducting advanced, large-scale, multicenter RCTs to tackle all the concerns related to assessing whether there are any statistically and clinically significant changes. Furthermore, to confirm the beneficial effect of GLN on the functions of different body organs, including the kidney, liver, and heart, particularly in burn patients.

## Conclusion

This systematic review and meta-analysis of seven randomized clinical trials determined that the risk of suspected infection was lower in GLN-receiving patients. At the same time, there was no significant difference in confirmed infections and LOH between the two groups. After excluding two studies, the risk of death was lower in the GLN-receiving group. However, there were no significant differences in the duration of mechanical ventilation between the two groups. GLN supplementation is beneficial for burn patients in decreasing the risk of suspected infection and death. Further prospective studies are needed to confirm the beneficial effect of GLN on the functions of different body organs.

## Declaration of Competing Interest

The author(s) declare no potential competing of interest with respect to the research, authorship, and/or publication of this article
